# Oxidative Power: Tools for Assessing LPMO Activity on Cellulose

**DOI:** 10.3390/biom11081098

**Published:** 2021-07-26

**Authors:** Federica Calderaro, Loes E. Bevers, Marco A. van den Berg

**Affiliations:** 1 DSM Biotechnology Center, 2613 AX Delft, The Netherlands; loes.bevers@dsm.com (L.E.B.); marco.berg-van-den@dsm.com (M.A.v.d.B.); 2 Molecular Enzymolog y Group, University of Groningen, Nijenborgh 4, 9747 AG Groningen, The Netherlands

**Keywords:** lytic polysaccharide monooxygenase, enzyme assay, cellulose, lignocellulosic biomass, chromatography, enzyme kinetics

## Abstract

Lytic polysaccharide monooxygenases (LPMOs) have sparked a lot of research regarding their fascinating mode-of-action. Particularly, their boosting effect on top of the well-known cellulolytic enzymes in lignocellulosic hydrolysis makes them industrially relevant targets. As more characteristics of LPMO and its key role have been elucidated, the need for fast and reliable methods to assess its activity have become clear. Several aspects such as its co-substrates, electron donors, inhibiting factors, and the inhomogeneity of lignocellulose had to be considered during experimental design and data interpretation, as they can impact and often hamper outcomes. This review provides an overview of the currently available methods to measure LPMO activity, including their potential and limitations, and it is illustrated with practical examples.

## 1. Introduction

**Figure 1:**
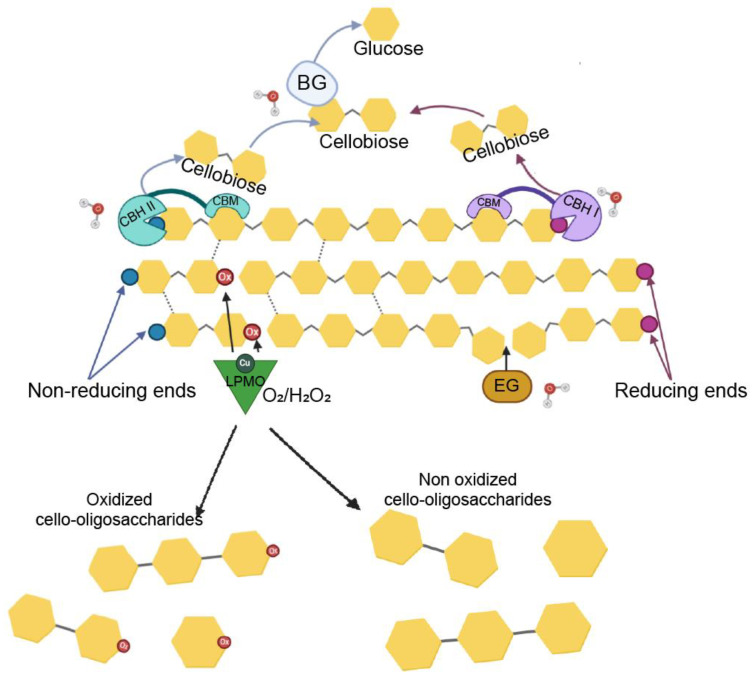
Schematic representation of enzymes acting in the deconstruction of cellulose. LPMOs (C1-oxidizing: EC 1.14.99.54; C4-oxidizing: EC1.14.99.56) use either O_2_ or H_2_O_2_ to hydrolyze cellulose chains in the crystalline regions, making the cellulose more accessible for the action of hydrolytic enzymes. Endoglucanases (EG; EC 3.2.1.4) hydrolyze the glycosidic bond in the amorphous regions of the cellulose chains, generating new reducing and non-reducing ends. Cellobiohydrolases (EC 3.2.1.91) processively release cellobiose units from the reducing (CBH I) and non-reducing (CBH II) ends of the cellulose chains. Cellobiose is then converted into glucose units by β-glucosidases (BG; EC 3.2.1.21). Ox, oxidated glucose moiety; CBM, carbohydrate binding module.

**Figure 2:**
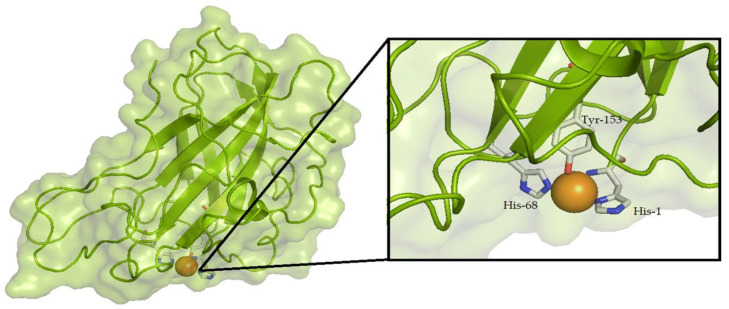
Crystal structure and catalytic center of a fungal LPMO. Schematic representation of the crystal structure and a close-up view of the histidine-brace of the cellulose-active *Tt*LPMO9E from *Thielavia terrestris* (UniProt ID G2RGE5, PDB 3EJA, strictly C1-oxidizing). The copper atom is shown as an orange sphere. The residues involved in the coordination of the copper are shown as sticks.

Until recently, the enzymatic conversion of recalcitrant polysaccharidic materials such as chitin or cellulose was thought to solely depend on the activity of hydrolytic enzymes. The discovery of lytic polysaccharide monooxygenases (LPMOs) has revolutionized this view [1]. LPMOs are copper-containing enzymes that boost the activity of the classical hydrolytic enzymes on either chitin [2] or cellulose [3]. In 2010, Vaaje-Kolstad et al. [1] showed that LPMO’s catalytic mechanism is based on an oxidative reaction, which results in the cleavage of glycosidic bonds in polysaccharides, and an overall disruption of the substrate’s structure, facilitating the activity of hydrolytic enzymes such as endoglucanase (EG), cellobiohydrolase (CBH), and β-glucosidase (BG) (Figure 1). LPMOs are now classified as auxiliary activities (AA) [4,5] and grouped into seven families in the CAZy database (CAZy. Available online: http://www.cazy.org/, accessed on 12 April 2021). Enzymes belonging to families AA9 and AA10, originally known as GH61 and CBM33, respectively, were the first to be characterized [1,6]. AA9s include fungal enzymes that are active on different substrates, such as cellulose [6,7,8], soluble cello-oligosaccharides [9], xylan [10], hemicelluloses [11,12,13], and starch [14,15]. AA10 LPMOs, found in all domains of life but mainly in viruses and bacteria, have been reported to have activity on chitin and cellulose [1,16]. AA11, AA13, and AA14 LPMOs are only found in fungi and show activity towards chitin, starch, and cellulose-bound xylan, respectively [14,17,18]. Members of the AA15 family, found in Eukarya and viruses, show activity on both chitin and cellulose [19]. The most recently described family is AA16, with one enzyme so-far characterized showing activity on cellulose [20]. One peculiar structural feature of LPMO is the presence of a flat binding surface in which two conserved histidines coordinate the Cu^2+^ ion and form the so-called histidine brace [6,21,22]. The third residue coordinating the copper is a tyrosine for AA9 (Figure 2), as well as for AA11/13/14/15 [18], and a phenylalanine for most of the AA10 [23]. The reduction of Cu^2+^ to Cu^+^ is driven by an external electron donor, which can be a small molecule such as ascorbic acid or certain phenols [1,24,25], biomass components such as lignin and its derivatives [26,27], an enzyme such as cellobiose dehydrogenase and pyrroloquinoline quinone (PQQ)-dependent pyranose dehydrogenase (PDH) [28,29,30,31,32,33], or light [34,35,36]. This represents the first step in the oxidative reaction of LPMO. Following this, a reaction with O_2_ [1] or H_2_O_2_ [30] leads to the hydroxylation of either the C1 (resulting in the formation of an aldonolactone) or C4 carbon (with the formation of a 4-ketoaldose) of the glucose moieties in the polysaccharide substrate [10,11], releasing both soluble and insoluble oxidized products [6]. The majority of LPMOs characterized so far, according to the CAZy database (CAZy. Available online: http://www.cazy.org/, accessed on 12 April 2021) [5], are C1- or C1/C4-oxidizing LPMOs, with fewer strictly C4-oxidizers reported in literature. Over the past few years, several studies have focused on the identification of elements involved in LPMO regioselectivity because the sequence-based unambiguous prediction of C1/C4-oxidizing activity is not as straightforward. In this regard, the precise positioning of the active site copper towards the substrate [37,38,39,40], which is influenced by the local amino acid configuration, appears to be the main determinant.

Since their discovery, LPMOs have attracted attention from both the academic sector and industry. The ability of cellulose-active LPMOs to improve the hydrolysis efficiency of classical cellulases on different lignocellulosic materials represents an interesting option for industries to reduce the enzyme loadings needed in the process of biomass to biofuel conversion [41,42]. Despite extensive research conducted in the last few years, accurately measuring and quantifying LPMO activity is still a difficult task, partly due to the insoluble nature of the substrates. As a result, less than 1% of the ~8500 known LPMO enzymes (CAZy. Available online: http://www.cazy.org/, accessed on 12 April 2021) are biochemically characterized. With several key questions still to be elucidated (e.g., inactivation mechanisms, reducing agents, and effects of O_2_, H_2_O_2_, or electron acceptors under industrial conditions), there is a need for a variety of analytical methods. The most commonly applied method is to assess LPMO activity by analyzing the oxidized oligosaccharides released after the incubation of the enzyme with the substrate through high performance anion exchange chromatography (HPAEC) and/or mass spectrometry [1,16]. Though very sensitive, these methods can be quite time-consuming and require specialized equipment that is not available in every facility. Despite several studies focusing on quick and easy methods to assess LPMO activity, we are still lacking a screening method to compare the oxidative activity of LPMO on different polysaccharides. The aim of this review is to give an overview of available methods that have been used to assess LPMO activity, with their advantages and drawbacks.

## 2. Analytical Methods

**Figure 3:**
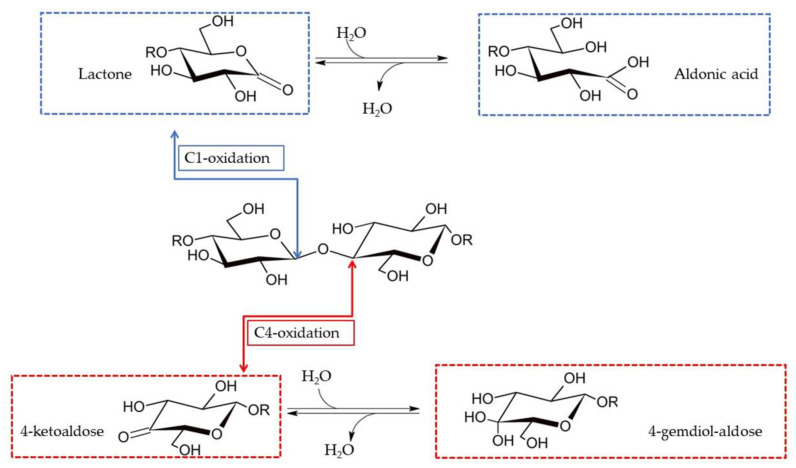
LPMO activity on cellulosic substrates. Oxidation can occur either at the C1 or C4 positions, leading to the formation of a lactone or a 4-ketoaldose, respectively.

LPMOs act on the glycosidic bonds of cellulose chains by using O_2_/H_2_O_2_ and electrons to extract a hydrogen atom from the C1 or C4 carbon and form a radical that is immediately hydroxylated. This species is unstable, leading to the breakage of the glycosidic bond and the oxidation of the chain end [9,28]. As reported extensively, depending on the type of LPMO, the oxidation can take place either at the C1 (EC number: EC 1.14.99.54) or C4 carbon (EC number: EC 1.14.99.56) [1,7,39,43,44]. C1-oxidation results in the formation of an oligosaccharide with a regular non-reducing end and a lactone group on the other end that is hydrated to its aldonic acid form. C4-oxidation results in an oligo with a regular reducing end and a 4-ketosugar on the other end that is then hydrated to gem-diol (Figure 3). The most common identification and quantification methods for LPMO products rely on chromatographic methods and mass spectrometry analysis, which differ depending on the type of LPMO–substrate combinations to analyze. Oxidized cello-oligosaccharides can be successfully separated by means of high performance anion exchange chromatography (HPAEC) [1,43], and they can be analyzed by different detection methods (pulsed amperometric detection (PAD), electrospray-ionization mass spectrometry (ESI-MS), and charged aerosol detection (CAD)); see below for more details. In parallel, MALDI-TOF MS is normally employed to annotate the oxidized products detected with HPAEC. Another chromatographic method successfully applied to separate C1- and C4-oxidized cello-oligosaccharides is porous graphitized carbon (PGC) chromatography, either alone or in combination with CAD [45].

### 2.1. HPAEC-PAD

**Figure 4:**
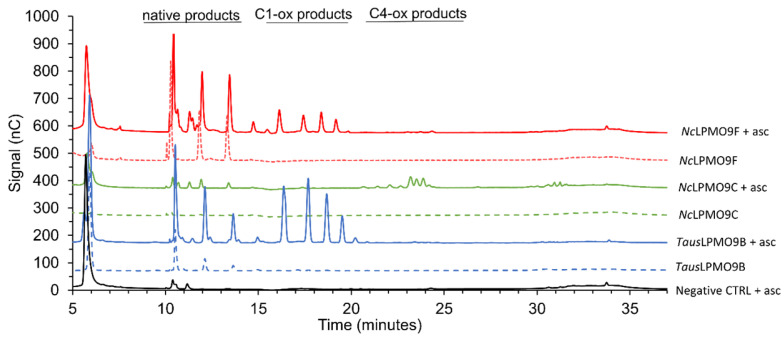
Products released from cellulose by different AA9s [47]. Strictly C1-oxidizing LPMOs (*Taus*LPMO9B, blue line; *Nc*LPMO9F, red line) and strictly C4-oxidizing LPMO (*Nc*LPMO9C, green line) in the presence (solid lines) or absence (dotted lines) of ascorbic acid (asc). The black solid line represents the negative control containing all components of the reaction mixture except for the enzyme.

The most commonly used quantitative method to assess LPMO activity is measuring the amount of products released after incubation by means of high-performance chromatography, particularly HPAEC with PAD [43]. A typical elution pattern is presented in Figure 4: native (non-oxidized) products elute first, followed by C1- and C4-oxidized products. Double-oxidized products normally elute later in the gradient. Here, C1-oxidizing *Nc*LPMO9F [46] and *Taus*LPMO9B [47] and C4-oxidizing *Nc*LPMO9C [46] were incubated with phosphoric acid swollen cellulose (PASC) in the presence of ascorbic acid as an electron donor. In order to separate the oligosaccharides present in the reaction mixtures, alkaline pH is applied during the elution. This is particularly favorable for the separation and detection of C1-oxidized products, which are negatively charged at alkaline pH, thus allowing for separation from native products. On the other hand, at alkaline pH, C4-oxidized products tend to be subjected to chemical modifications such as tautomerization [9] and on-column decomposition [45]. Due to this and the late elution of the products in a phase when the percentage of acetate in the mobile phase is high and more likely to suppress other signals, the intensity of the C4 peaks is relatively low compared to C1 peaks. On top of this, C4-oxidized products in alkaline environments tend to get converted to native (non-oxidized) products, which can explain the increase in native products released upon the incubation of substrates with C4-oxidizing LPMOs [45,48]. Another drawback is that due to the high pH and salt concentrations used for separation, the MS analysis of HPAEC samples is not straightforward. Coupling the HPAEC-PAD set-up to an ion trap MS with online anion suppression has been proven to be an effective tool for the direct annotation of C1-oxidized products but does not allow for the detection of masses corresponding to C4-oxidized products or C1/C4-oxidized products [45].

**Figure 5:**
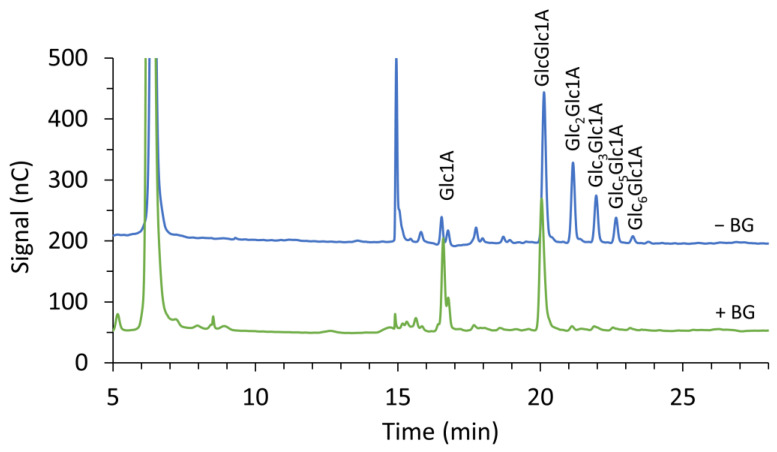
Products released from cellulose by *Taus*LPMO9B [47] before (blue line) and after (green line) treatment with BG; 1 µM *Taus*LPMO9B was incubated with 0.1% (*w*/*v*) PASC and 1 mM ascorbic acid in a 50 mM sodium acetate buffer pH 5 at 45 °C for 16 h. Reaction mixtures were centrifuged and filtered before the treatment of soluble products with 1 unit of BG from almonds (Sigma, St Louis, MO, USA) at 37 °C for 16 h in a 50 mM sodium acetate buffer pH 5. Glc1A, gluconic acid; GlcGlc1A, cellobionic acid; Glc2 Glc1A, cellotrionic acid; Glc3Glc1A, cellotetraonic acid; Glc5Glc1A, cellohexaoinic acid; Glc6Glc1A, celloeptaonic acid.

The quantification of released soluble products requires a comparison to oligosaccharide standards of known concentration. To “simplify” the product profile and allow for easier quantification, the reaction mixtures can be treated with different enzymes. Treatment with a GH5 endoglucanase, such as *Tf*Cel5A [49], degrades longer oxidized products to a mixture of oxidized trimers and dimers [23,35,38,50,51]. Standards for these trimers and dimers can be produced by incubating cellotriose and cellobiose, respectively, with cellobiose hydrogenase *Mt*CDH [38]. In a similar approach, Frommaghen et al. [52] developed a method to quantify C1-oxidating activity by using BG to convert longer oxidized oligosaccharides into gluconic and cellobionic acid, which can be compared with commercially available standards (see Figure 5 in this work and Figure 1 in [52] for examples of the effect of BG on product profile). The quantification of C4-oxidation, on the other hand, is not as simple, due to the instability of these compounds during analysis and the lack of proper standards. Nevertheless, Müller et al. exploited the ability of a C4-oxidizing LPMO (*Nc*LPMO9C) to cleave cellopentaose and generate equimolar amounts of native cellotriose (commercially available) and C4-oxidized cellobiose (Glc4gemGlc); this mixture was used as an external standard to indirectly quantify the amount of Glc4gemGlc by comparing it with the native cellotriose compound [53]. Glc4gemGlc can subsequently be incubated with *Mt*CDH to generate a standard for the double-oxidized dimer (Glc4GemGlc1A) [23]. Because of the challenges faced when assessing LPMO activity, there are not many kinetic data available in the literature. Bissaro et al. reviewed the apparent LPMO rates that were either published or calculated from available progress curves [54]. The highest oxidative rates have been reported for reactions with H_2_O_2_ [30,55], followed by reactions fueled by photocatalytic systems [34,56].

When measuring the activity of an isolated LPMO, the above-mentioned methods are suitable for the detection of small soluble products only. Considering that a fraction of oxidized LPMO products still remains in the insoluble fraction, reported LPMO activities are usually underestimated. A way to address this problem is by degrading the insoluble fraction of the LPMO–substrate mixture using hydrolytic enzymes, resulting in the release of residues that can be quantified by chromatographic methods. Applying this principle during the characterization of the cellulose-active *Tt*LPMO9E, Cannella et al. were able to quantify the total cellulose oxidation by measuring the amount of glucose and gluconic acid released after the treatment of LPMO reaction products with a commercial cellulosic cocktail [34]. The same method was applied by Frommhagen et al. [52], who showed that the amount of oxidized oligosaccharides was higher in the insoluble fraction during the early hours of incubation compared to the soluble fraction. Based on the progress curve reported in the latter study, the total amount of the released oxidized products can be estimated as about two times higher than the soluble oxidized products, with values in the range of, respectively, 28 and 15 µM after 8 h of enzyme incubation. Though enzymatic hydrolysis is often not 100% complete due to various limiting factors, the only alternative is complete acid hydrolysis, but this destroys the oxidative moieties. HPAEC-PAD is powerful tool that is also routinely applied for the identification of products released from other substrates, such as xyloglucan [11,48,52,57,58].

Though very sensitive, the methods described above come with certain limitations. Due to long analysis times (typical incubation times go up to 24 h and approximately 30 min per chromatographic run), it is not viable to monitor LPMO activity during production (i.e., fermentation and purification). On top of that, the limitation of the analysis to solubilized products and the lack of readily available reference compounds (the synthesis of which can become laborious and costly) are further complications.

### 2.2. Porous Graphitized Carbon (PGC) Chromatography

PGC chromatography has proven to be an efficient method for the separation of native and oxidized cello-oligosaccharides. An important advantage of this method is the use of a buffer compatible with MS analysis (50 mM ammonium bicarbonate at pH 8). The PGC column separates compounds based on the surface contact between the molecular area of the analyte and the graphite surface, as well as the polarity of the analyte itself [59]. Because of their different p*K*_a_, aldonic acids and native products behave differently at the slightly alkaline conditions (pH 8) used during PGC chromatography, with the former being negatively charged (e.g., p*K*_a_ of cellobionic acid = 3.51 [45] and p*K*_a_ D-gluconic acid = 3.7 [60]) [43]. The protocol developed for the separation of aldonic acids has also proven to be successful for the simultaneous analysis of C1- and C4-cellodextrins, particularly for the separation of gem-diol and aldonic acids with the same DP [43]. The resolution decreases for mixtures of native and C4-cellodextrins or aldonic acids and double-oxidized species, as their retention times are similar and they tend to co-elute. The absolute identification of these products therefore requires either MS detection (see Figure 4 in [45] for an example of porous graphitized carbon chromatograms of either native, C1-, C4-, or double-oxidized cellodextrins) or β-glucosidase treatment to remove native and C1-oxidized species [45]. Moreover, the strong affinity of the PGC column stationary phase for longer oligosaccharides does not allow for the separation of native-oligosaccharides with a DP above five. Combining PGC with CAD enables product quantification. This is particularly effective for C4-cellodextrins, with amounts as low as nanograms detectable when using low ionic strength eluents, therefore making this method sensitive enough to be used in kinetic studies [45].

### 2.3. RP-UHPLC-UV-ESI-MS/MS

In 2017, Frommhagen et al. developed a protocol for the separation of C4-oxidized oligosaccharides based on reverse phase-ultra high performance liquid chromatography (RP-UHPLC) paired to non-reductive 2-aminobenzamide (2-AB) labeling [61]. Reductive labeling via amination had already been used before for the identification of gluco-oligosaccharides [62]. The process is based on the reaction of the reducing end of the oligosaccharide with a fluorophore–amine complex. This reaction results in the generation of unstable intermediates, such as imines, which are converted into more stable amines using reducing agents. The reductive labeling of C4-oxidized products derived from LPMO reactions can be challenging because the ketone group tends to also be reduced in the presence of strong reducing agents [63]. Building on this knowledge, Frommhagen and co-workers developed a non-reductive labeling method. The new method resulted in the successful identification of native and C4-oxidized products without the use of strong reducing agents. The advantage of this approach is, as for the PGC method, the improvements in the analysis and identification of native and C4-oxidized oligosaccharides, for which HPEAC separation is not efficient. In contrast to PGC, the use of non-reductive labeling allows for the use of non-buffered eluents, which are less detrimental in terms of mass spectrometry sensitivity, for separation.

### 2.4. UHPLC-ESI-MS

The separation of polar compounds, such as LPMO products, can be efficiently achieved with UHPLC. Though not routinely used, the combination of UHPLC with ESI-MS can complement MALDI-TOF analysis in getting a complete spectrum of product profile. Since the 2,5-dihydroxybenzoic acid (DHB) matrix generates signals in the low molecular weight region of the spectrum, the optimal detection range for MALDI-TOF MS includes longer oligosaccharides (DP between 3 and 10). By combining the high sensitivity offered by ESI-MS with the efficiency of hydrophilic interaction chromatography to analyze polar compounds, De Oliveira Gorgulho Silva et al. developed a UHPLC-ESI-MS method that can detect smaller oligosaccharides (DP 1–5) [64]. Additionally, this method is compatible with online mass-spectrometry detection.

### 2.5. MALDI-TOF-MS

A method to qualitatively assess the products released by LPMO, after incubating the enzyme with substrate and reducing agents, is MALDI-TOF MS [1,6,65,66]. Even though the 4-ketosugars and lactones have identical masses (*m*/*z* −2 compared to the corresponding native product), it is still possible to discriminate them by means of MS [65,66]. At the neutral pH used during analysis, the equilibrium between the lactone and its aldonic acid form is shifted towards the latter. Because of its carboxylic group, the aldonic acid is more prone to form double salt adducts. In contrast to the C1 products, the 4-keto-sugar generated through C4-oxidation and its hydrated gem-diol form are in equilibrium and typically result in single sodium adducts. Taken together, the detection of masses corresponding to the presence of two sodium ions is an indication of the presence of C1-oxidized products in the reaction mixture, while their absence suggests C4-oxidizing activity (see [23,67] for examples of the MALDI-TOF spectra of oxidized products). Additionally, the permethylation of released oxidized products allows for the unambiguous identification of C1-oxidation [68]: the conversion of hydrogen atoms to methyl groups leads to the generation of +30 Da species for products oxidized at the reducing end compared to the corresponding non-oxidized oligosaccharides [6,15,19]. The analysis of reaction products through MALDI-TOF has been successfully applied to characterize LPMO activity on several substrates. In addition to cellulose, MALDI-TOF is routinely applied to detect activity on chitin [1,67,69], hemicellulose [11,48,57,58,70,71], and starch [15]. For instance, by using this method, Lo Leggio et al. detected the aldonic acids released from starch by *An*(AA13), which could not be detected through chromatographic methods [15]. Though the MALDI-TOF-MS method is purely qualitative, it can be useful to assess the regioselectivity and activity of particular LPMOs.

### 2.6. NMR

Some studies have used nuclear magnetic resonance (NMR), in particular as a means to assess the possibility of C6-oxidation activity, as C6-oxidized products cannot be easily and unambiguously identified with mass spectrometry. NMR has been successfully used to identify the products released by *Nc*LPMO9C as C4-oxidizing products [9] and to confirm the absence of C6-oxidation. Westereng et al. [43] also used NMR to confirm the identity of a cellotrionic acid generated through semi-preparative PGC-LC, and they used it as standard for the assessment of chromatographic methods.

### 2.7. XPS

X-ray photoelectron spectroscopy (XPS) is a powerful surface technique that allows for elemental composition analysis. The irradiation of the sample with a beam of X-rays causes photoelectron emission from the surface, generating a specific kinetic energy that can be measured and used to identify the chemical composition of the sample surface.

As LPMOs modify the surface of the substrate, the introduction of oxygen atoms can be detected and analyzed by XPS. This method was successfully applied by Selig and co-workers [72] in the evaluation of *Sc*LPMO10C’s influence on the interactions between cellobiohydrolase and cellulose. In this case, XPS was used to verify the presence of oxidized cellulose on cellulose-coated SiO_2_ sensors caused by the LPMO treatment. Though powerful, this costly method has not been so widely applied. Vuong et al. used it as a comparison to validate their newly developed method [73] (see section “Carbodiimide conjugation of carboxyl groups”).

## 3. Methods Based on Absorbance/Fluorescence

**Figure 6:**
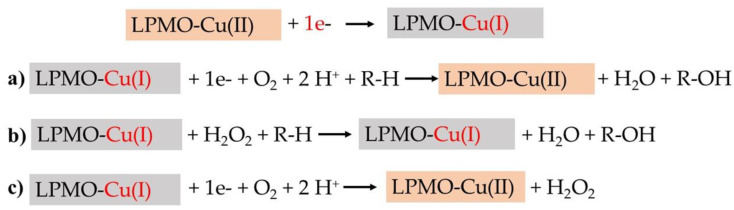
LPMO reaction mechanism. The first step is the reduction of the Cu(II) in the catalytic center by one electron to generate an LPMO-Cu(I) species. In the presence of a substrate (R-H), LPMO can react either with (**a**) O_2_ or (**b**) H_2_O_2_, leading to substrate hydroxylation (R-OH). In the absence of a substrate (**c**), oxygen reduction by LPMO results in the generation of H_2_O_2_.

Though very sensitive, chromatographic methods can be time-consuming and requires high-end instrumentation. Therefore, efforts have been made towards the development of alternative fast and easy assays that can be used to measure and/or characterize activity by making use of different LPMO properties. In contrast to directly measuring reaction products, LPMO activity can also be indirectly determined by measuring, for instance, side products. As described above, LPMOs are oxidative enzymes with a Cu(II) atom in the catalytic center. The reaction starts with the reduction of Cu^2+^ to Cu_+_ by an external electron donor. The reduced copper then reacts with the co-substrate, originally proposed to be O_2_, forming an activated copper–oxygen complex that enables the oxidation of the substrate and the cleavage of the glycosidic bond (Figure 6; [1,29]). A recent study proposed H_2_O_2_ as a kinetically relevant co-substrate for LPMO [30]. In this proposed mechanism, after the reduction of Cu^2+^ to Cu^+^ and in the presence of a substrate, LPMO reacts with H_2_O_2_, leading to the abstraction of a hydrogen atom from the substrate and the hydroxylation of the latter [30,74] (Figure 6b). In the absence of a substrate, the activated copper–oxygen-complex undergoes a so-called “uncoupling reaction,” leading to the generation of H_2_O_2_ (Figure 6c).

### 3.1. AmplexTM Red/Horseradish Peroxidase

**Figure 7:**
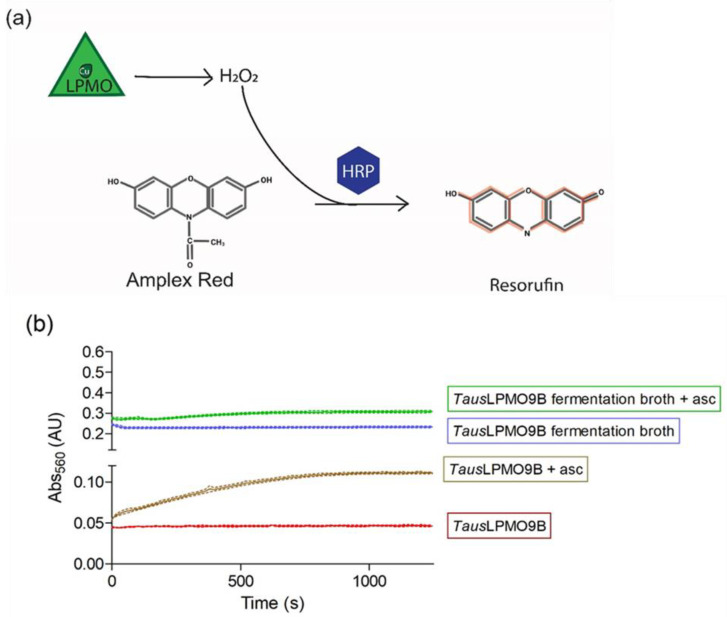
Amplex^TM^ Red assay used to detect LPMO activity. (**a**) Horseradish peroxidase (HRP) utilizes the H_2_O_2_ generated by LPMOs to convert Amplex^TM^ Red to resorufin, a fluorescent molecule with excitation and emission maxima at 571 and 585, respectively. (**b**) *Taus*LPMO9B activity tested with the Amplex^TM^ Red assay before (green and blue lines) and after (brown and red lines) enzyme purification. Reactions were run in 50 mM sodium phosphate buffer pH 7 and contained 5 U/mL of HRP, 100 µM Amplex^TM^ Red, and 20 µL of purified *Taus*LPMO [47] or diluted fermentation broth (1:4) in a final volume of 100 µL. Reactions were started by the addition of ascorbic acid (brown and green lines) to a final concentration of 50 µM. Reactions were run in triplicate; the dotted lines represent the standard deviation of 3 independent replicates. Various dilutions were tested for the fermentation broth, but the background absorbance was always too high to allow for the clear detection of LPMO activity. The LPMO content of fermentation broth was measured by the quantification of the protein band after SDS-PAGE electrophoresis. The intensity of the band was measured and compared to a BSA standard curve by digital imaging using ImageJ (Image Processing and Analysis in Java; available at http://imagej.nih.gov/ij/, accessed on 5 January 2021). Samples were diluted to fit in the linear quantifiable range (0.050–0.250 mg/mL protein). The LPMO content of the fermentation broth was estimated to be 0.1 mg/mL.

The H_2_O_2_ produced by the “uncoupling reaction” can be quantified by the Amplex^TM^ Red/Horseradish peroxidase (HRP) assay [46]. HRP can oxidize Amplex^TM^ Red using H_2_O_2_ to resorufin in a 1:1 stoichiometry (Figure 7a). This product is a red fluorescent compound with an absorption maximum at 536 nm and an emission maximum at 587 nm [75]. Because it is quick and easy to apply, the Amplex^TM^ Red assay has been widely used to check for LPMO activity [12,46,76,77]. Despite its widespread use, it comes with some disadvantages including a low sensitivity and a requirement of high amounts of LPMO (3 µM). On top of that, the presence of metal ions in fermentation media can interfere with the assay, as shown for *Taus*LPMO9B (Figure 7b). In [47], this enzyme was produced in *A. niger* grown in corn steep liquor (CSL). When fermentation broth was directly tested, the background absorbance values were too high to allow for the clear detection of the LPMO activity (Figure 7b, blue line). Another aspect to consider when using this assay is the amount of H_2_O_2_ generated by the reaction of the reductant with oxygen. Stepnov et al. compared the effects of different amounts of gallic or ascorbic acid, highlighting the striking effect that free copper in LPMO preparations can have on the determination of LPMO rates [78]. 

Nevertheless, this method is very useful to check for LPMO activity in more “clean” samples (e.g., during and after purification).

### 3.2. Nickel/Pyrocathecol Violet

Another rapid method to measure the LPMO-dependent oxidation of insoluble substrates was proposed by Wang et al. for cellulose and chitin-active LPMOs [79]. The principle of this ion adsorption/desorption method relies on the fact that the aldonic acids generated though C1-oxidation are negatively charged. Briefly, LPMO-treated polysaccharides are centrifuged to remove solubles. The insoluble polysaccharides are then incubated with Ni^2+^, which is adsorbed by the carboxyl groups generated by LPMO reaction. The concentration of adsorbed Ni^2+^ can easily be quantified using pyrocatechol violet (PV), a complexometric indicator that chelates the cation and absorbs at 650 nm. Therefore, by measuring the amount of unbound Ni^2+^ present in the supernatant, it is possible to quantify the amount the carboxylate moieties adsorbed by the polysaccharide surface [79]. The relative simplicity and rapidity of this method (multiple measurements can be done at the same time in a 96-well plate) make it an interesting option for comparing different LPMOs. However, the method is limited to the detection of C1-oxidized sites only and lacks a reliable standard that simulates the oxidation pattern introduced by LPMO on the polysaccharides. Nevertheless, the method was successfully used by Ni et al. to elucidate the role of a synthetic lignin polymer on the activity of an LPMO from *Pleurotus ostreatus* (*Pc*LPMO9D) and, in particular, the inhibition effect of high amounts of LPMO on cellulase-mediated hydrolysis [80].

### 3.3. D-Gluconic Acid/D-Glucono-d-Lactone Assay

Unfortunately, most of the methods listed thus far for the detection of LPMO products are limited to clean, model cellulosic substrates. The need for a simple method for the quantification of oxidizing activity, also on more complex lignocellulosic substrates, was therefore urgent. This was the focus of a recent study by Keller et al., who developed a quick approach for the quantification of gluconic acid in both microcrystalline cellulose and pretreated wheat straw [81]. The treatment of LPMO C1-oxidized products with BG leads to the hydrolysis of the products and the generation of glucose and gluconic acid, among others. The latter could easily be spectrophotometrically quantified with a D-Gluconic acid/D-Glucono-d-lactone assay kit (Megazyme). In this assay, gluconic acid is converted by gluconate kinase in D-gluconate-6-phosphate, which is converted to D-gluconate-6-phosphate by 6-phosphogluconate dehydrogenase in the presence of NADP. The NADPH formed in this reaction can be quantified at 340 nm and is in a stochiometric 1:1 ratio with gluconate. This method can be applied to quantify gluconic acid formation in both soluble and insoluble products. For the latter, an additional CBH I hydrolyzation of the insoluble reaction pellet is required prior to BG treatment. All reagents are commercially available, and the relative simplicity of the method enables automatization (96 samples in the MTP format in 30 min) and the application on both model substrates, as well as lignocellulosic material. its main limitation is that it only is suitable for C1-oxidizing LPMOs.

### 3.4. 2,6-Dimethoxyphenol

**Figure 8:**
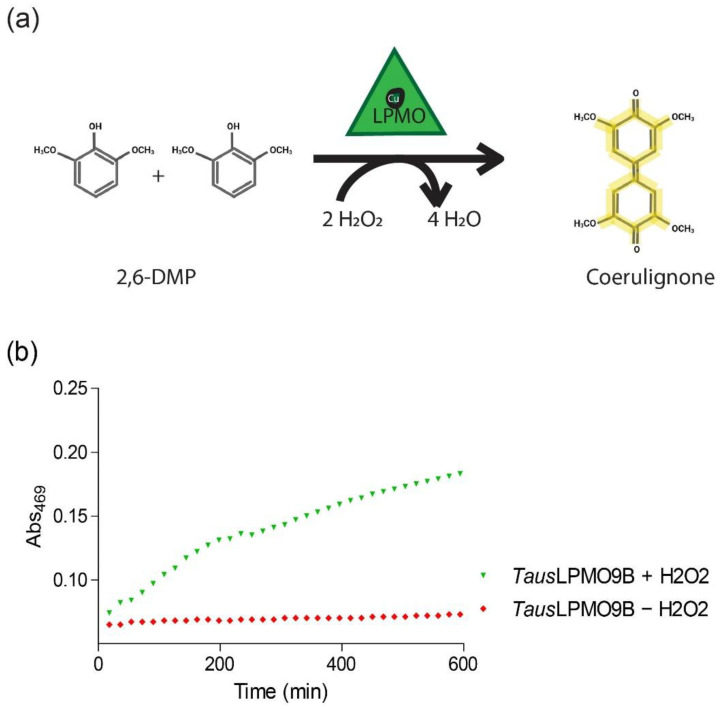
DMP assay to measure LPMO activity. (**a**) In the presence of H_2_O_2_, LPMOs catalyze the conversion of 2,6-DMP to coerulignone, a chromogenic compound with a maximum absorption at 469 nm. (**b**) *Taus*LPMO9B [47] activity with DMP in the presence (green) or absence (red) of H_2_O_2_. Reactions were run at 30 °C and contained 0.06 mg/mL purified LPMO, 10 mM substrate, and 1 mM H_2_O_2_ in a 100 mM sodium acetate buffer pH 4.5.

Exploiting the recently discovered role of H_2_O_2_ in the LPMO reaction [30], Breslmayr et al. [82,83] developed a spectrophotometric method for quick activity assessment. This assay is based on the oxidation of 2,6-dimethoxyphenol (2,6-DMP) by LPMO peroxidase activity, resulting in the generation of a 2,6-DMP phenoxy radical (Figure 8). Two 2,6-DMP radicals dimerize to hydrocoerulignone, which LPMO can convert to coerulignone, a chromogenic product that absorbs at 469 nm with a molar absorption coefficient of 53,200 M^−1^cm^−1^. The DMP assay has since then been widely used to assess LPMO activity [84,85,86], but one must consider that individual LPMOs have different sensitivities towards H_2_O_2_ [30], which may influence the efficiency of the assay. Nevertheless, the rapidity and simplicity of this method has made it a valuable tool for the quick assessment of LPMO activity in many studies.

### 3.5. Azo-Xyloglucan Assay

**Figure 9:**
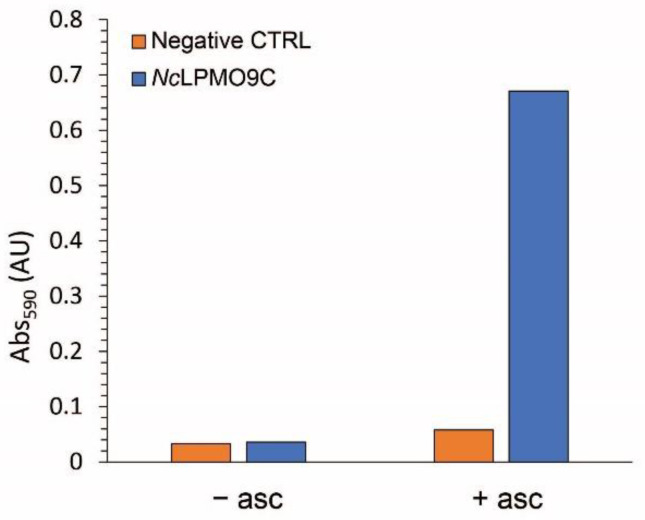
Azo-xyloglucan assay to measure LPMO activity. *Nc*LPMO9C activity with azo-xyloglucan in the presence or absence of ascorbic acid. Reactions were run at 55 °C for 10 min in a final volume of 0.6 mL and contained 0.06 mg/mL enzyme, 1% (*w*/*v*) substrate, 2.5 mM ascorbic acid in a 100 mM sodium acetate buffer pH 4.5. Reactions were stopped by the addition of 1 mL of 96% ethanol. After centrifugation at 1000× *g* for 10 min to separate the substrate, the released azo-labelled oligos were quantified by measuring absorbance at 590 nm.

Another fast method is based on the use of a soluble, dyed substrate, namely azo-xyloglucan (Megzayme) [87]. This substrate has previously been used for the evaluation of different endoglucanases, such as xyloglucan-specific [88,89] or GH7 endo-1,4-β-glucanases [90]. Due to their endo-mechanism of depolymerization, these enzymes release low-molecular-weight dyed components from the starting material. After the addition of a precipitant, such as ethanol, the remaining longer polysaccharides can be removed while the released and colored oligosaccharides remain in solution. As the substrate is dyed with Remazol Brilliant Blue R (RBBR), the color can be quantified by measuring the absorbance at 590 nm. The same assay can be used to evaluate LPMO activity by exploiting their need for reductants to fuel their reaction. In the absence of a reductant, LPMOs are not able to cleave the substrate, so no color can be detected in the supernatant after precipitation. In the presence of a reducing agent, such as ascorbic acid, LPMOs cleave the azo-xyloglucan substrate and release dyed oligosaccharides into solution. As this method has not been extensively applied, there are not much data to fully value its merits and/or limitations. The most straightforward assumption is that this method is only applicable for xyloglucan-active LPMOs, i.e., *Nc*LPMO9C (Figure 9). Additionally, the structure of the modified substrate, with about one dye molecule every 20 sugar residues, could hamper LPMO’s ability to cleave it. Therefore, it is not recommended to use this assay for the absolute comparison of different LPMOs. Nevertheless, it represents an easy and quick method to serve different purposes, such as the identification of LPMO activity in fermentation broth or during purification.

### 3.6. Reduced Phenolphtalein (rPHP) Assay

A new high-throughput colorimetric method based on the production of phenolphthalein (PHP) was recently developed by Brander and coworkers [91]. The assay relies on LPMO’s ability to produce PHP, a pink dye that can easily be quantified by measuring its absorbance at 552 nm, by oxidation of its reduced form (rPHP) in a mechanism that uses dehydroascorbate (DHA) as a co-substrate. Interestingly, rPHP oxidation is not boosted by ascorbic acid, the most commonly used electron donor for LPMO reactions, and actually causes the degradation of the enzyme in the reaction mixture. [91]. Considering that DHA is derived from the oxidation of ascorbate, it is not unlikely that it will be present in reactions with ascorbic acid, where it could also act as an electron donor for LPMOs. One disadvantage of the assay is its limitation to cellulose- and starch-active LPMOs (AA9 and AA13, respectively), and only limited DHA-dependent rPHP oxidation was shown for two chitin-active AA10s [91]. Nevertheless, the rPHP assay has several advantages over other assays, leading to the identification of new LPMO co-substrates, DHA and fructose (confirmed by cellulose cleavage). The method is also less sensitive to free copper in enzyme preparations compared to assays using ascorbate as a co-substrate. Moreover, reaction conditions reproduce what might happen in industrial settings due to the relatively slow LPMO-driven rPHP oxidation and lower sensitivity to O_2_ and H_2_O_2_ levels [91].

## 4. Other Methods

### 4.1. SYTO-62 Labeling of Carboxyl Groups

Another approach for the detection of LPMO products is labelling the oxidized sites generated on insoluble substrates. This was used by Eibinger et al. [92] to characterize the changes caused by LPMO on the surface of cellulose: the LPMO-treated substrates are incubated with a fluorescent probe reacting to carboxyl groups (SYTO-62) and analyzed by confocal laser scanning microscopy. With this approach, the authors were able to show that *Neurospora crassa Nc*LPMO9F mainly acts on the outer surface of cellulose and with multiple site attacks.

### 4.2. PACE and FRET

Polysaccharide analysis using carbohydrate gel electrophoresis (PACE) is a sensitive method for the analysis of polysaccharide structures that has also been used in the LPMO field [6]. It relies on two steps: first, derivatization of the reducing ends of sugars with a fluorophore and second, polyacrylamide-gel electrophoresis [93]. It was successfully applied by Frandsen et al. [22] to analyze the reaction products generated from PASC and oligosaccharides by a LPMO from *Lentinus similis* (*Ls*AA9). In this study, the reaction products were reductively aminated with 8-aminonaphthalene-1,3,6-trisulfonic acid (ANTS) before analysis. The results confirmed the activity of *Ls*AA9 on PASC, with the release of cellobiose and cellotriose (among others), and, importantly, they showed the activity of *Ls*AA9 on soluble oligosaccharides [22]. Exploiting the ability of this LPMO to act on soluble oligosaccharides, in the same work, the authors used fluorescence-labeled cellotetraose to determine the kinetic parameters of the oxidative reaction by LPMO. The cleavage of this derivatized substrate separates the quencher from the fluorophore, causing a strong fluorescence that can be quantified. Using this fluorescence resonance energy transfer (FRET) technique, the authors were able to determine a *K*_m_ of 43 and a kcat of 0.11 for *Ls*AA9 on cellotetraose. Despite its advantages, such as its relative simplicity, the FRET method its limitations. In addition to its high cost and laborious substrate preparation, its applicability is limited to soluble oligosaccharides, so it is not suitable for LPMOs only acting on insoluble substrates.

### 4.3. Carbodiimide Conjugation of Carboxyl Groups

A high-throughput screening method for assessing the activity of C1-oxidizing LPMO on insoluble substrates was developed based on the conjugation of the newly generated carboxylic acid groups through a carbodiimide (1-ethyl-3-[3-(dimethylamino)propyl]carbodiimide (EDAC)) to an amine-based fluorophore (7-amino-1,3-naphtalene-disulfonic acid (ANDA)) [73]. The conjugated products can be excited at 310 nm, and the resulting emitted fluorescence can be measured at 450 nm. Combining this assay with the classical methods for the detection of soluble products, such as HPAEC, can give more detailed profiles of the products generated by LPMO.

### 4.4. Turbidimetric Assay

LPMO activity on PASC can be correlated to the optical density of the substrate solution [94,95]. Because PASC is an amorphous and insoluble substrate, it forms an opaque solution. As a result of LPMO-driven solubilization, a change in its optical density can be observed. Applying this concept, Hansson and co-workers were able to compare the cellulose-cleaving activity of *Hj*LPMO9A with its ΔCBM-mutant, showing a 50% decrease in solubilization activity for the latter. Building on this, Filandr and co-workers developed an assay for monitoring the time-dependent activity of cellulose-active LPMOs [96]. The constant monitoring of the substrate absorbance at 620 nm allowed for a comparison of different reductant and H_2_O_2_ rates on LPMO activity. In their study, the authors showed that all the reactions in the presence of H_2_O_2_ converged to similar OD values, and this could not be reverted by the addition of fresh PASC/reductant/H_2_O_2_. Therefore, they identified the limiting factors of the assay as the unbound enzyme being damaged and the limited number of binding sites on the substrate itself.

## 5. Discussion and Conclusions

The use of LPMO in second-generation bioethanol production has sparked much research on these enzymes. Out of all the methods listed in this work, MS and HPAEC analysis remain the most reliable way of assessing LPMO. By using established protocols, over the past few years, LPMO’s reaction mechanism and potential in polysaccharide degradation has been elucidated. As discussed in this review, one of the many challenges encountered in this line of work is finding the right tools to study LPMO activity. As these enzymes require electrons and O_2_/H_2_O_2_ to perform their catalytic action, one must pay attention to the conditions used in activity assays. As exhaustively explained by Eijsink et al. [97], side reactions can play a big role controlling product levels, so care needs to be applied regarding the availability of reaction components (e.g., H_2_O_2_, O_2_, or reductant), as well as potential enzyme inactivation. Particularly when assuming H_2_O_2_ as a co-substrate fueling LPMO reactions, H_2_O_2_ availability (either produced by reaction of O_2_ with reductant or by LPMO side reactions) is the rate-limiting factor in evaluating enzyme activity [30,54,74,98]. Factors that could influence data interpretation have to be considered during experimental design. When analyzing LPMO progress curves, one can estimate the catalytic efficiency of the enzyme. Bissaro et al. gave a complete overview of reported and deduced kinetic parameters [54]. The highest reaction rates have been reported for enzymes fueled by H_2_O_2_ as co-substrate: *Sm*AA10A was reported to have a catalytic efficiency (*k*_cat_/*K*_m_) of 106 M^−1^ s^−1^ [98], which is in the same range as those reported for other fungal enzymes such as peroxygenases AAP II (*Agrocybe aegerita* peroxygenase) with a *k*_cat_/*K*_m_ for H_2_O_2_ of 2.79 × 105 M^−1^ s^−1^ [99] or lignin peroxidase from *Phanerochaete chrysosporium* with a *k*_cat_/*K*_m_ for H_2_O_2_ between 6.9 and 10.1 M^−1^ s^−1^ [100]. Reactions run in the absence of H_2_O_2_, on the other hand, appear to be rather slow: *Sc*AA10C was reported to have an oxidative rate (*k*_cat_) of about 3.2 min^−1^ in the absence of H_2_O_2_ and a rate of 82.4 min^−1^ in the presence of 200 µM H_2_O_2_ [30]. Stepping-up from these “semi” controlled in vitro environments, monitoring reaction conditions in more complex systems such as enzyme cocktails on colored, viscous, and inhomogeneous lignocellulosic substrates becomes even more challenging. Hence, the optimization of reaction conditions to fully exploit LPMO oxidative power has proven to be difficult: the presence of different redox compounds in lignin-rich substrate makes it virtually impossible to control side-reactions in the presence, for instance, of externally supplied H_2_O_2_ [47,101], which could be also detrimental to the other hydrolytic enzymes. The quantification of LPMO products through HPAEC-PAD relies on the use of additional enzymes to simplify the product profile and generate standards of known concentration. It is important to note that, as spectrophotometric and fluorometric methods can suffer from interfering elements in enzyme preparations, chromatographic methods can also have their limitations in terms of background interference. While these methods are very effective in detecting LPMO products in more “clean” samples, such as products released from Avicel, they might suffer some drawbacks when analyzing more complex samples such as lignocellulosic biomass hydrolysates. In this regard, saccharification experiments are usually performed with commercial enzyme cocktails that contain a mixture of hydrolytic enzymes. Evaluating the effect of LPMO on the performance of a whole cocktail is not an easy task. When spiking single LPMO enzymes on top of an LPMO-poor cocktail, such as Celluclast^®^, an increase in glucose yield is observed and can be considered an “indirect measurement” of LPMO contribution [47,53,102,103,104]. More modern cellulolytic cocktails, such as Cellic^TM^ CTEC2 or CTEC3, contain LPMOs in addition to the classical hydrolytic enzymes. In this case, LPMO’s contribution can be evaluated by a comparison of saccharification yields run in the presence or absence of O_2_ [53,102]. The measurement of LPMO products in this type of set-up is not as straightforward. The BG present in enzyme cocktails is able to convert longer C1-oxidized products into gluconic acid [105], which elute early in the chromatographic analysis and can suffer from interference due to other overlapping peaks. On the other hand, Müller et al. showed that BG is not able to cleave C4-oxidized products [53]. As C4-oxidized cellobiose (Glc4gemGlc) elutes later in the gradient in an area where background interference is not too pronounced, it can be used to monitor LPMO activity during the saccharification of steam-exploded birch [53,102,106]. Glc4gemGlc is the only LPMO product identified when using Cellic^TM^ CTEC preparations, and its levels correlate with overall saccharification yields.

Though challenging, the characterization of LPMO activity has enabled tremendous progress in the elucidation of its reaction mechanism. More than 10 years after its discovery, LPMO is still object of extensive research. Though its unique oxidative power is being exploited in the biorefinery industry, there are also LPMOs that do not seem to be related to polysaccharide degradation but have a physiological role [107]. For example, some AA10s have been attributed a role as virulence factors in human infections caused by *Vibrio cholerae* [69,108], *Listeria monocytogenes* [109,110], and *Pseudomonas aeruginosa* [111,112]. The broad range of described tools and methods will certainly enable further studies and reveal additional functions of LPMO in nature.

## Data Availability

The data presented in this study are openly available for all figures and samples.

## Abbreviations

AA	Auxiliary Activity
BG	β-glucosidase
CAD	Charged Aerosol Detection
CAZymes	Carbohydrate Active enZymes
CBH	Cellobiohydrolase
CBM	Carbohydrate Binding Module
CDH	Cellobiose Dehydrogenase
CSL	Corn steep liquor
Glc1A	Gluconic acid
GlcGlc1A	Cellobionic acid
DHA	Dehydroascorbate
DHB	Dihydroxybenzoic Acid
DMP	Dimethoxyphenol
DP	Degree of Polymerization
FRET	Fluorescence Resonance Energy Transfer
Glc4GemGlc	gem-diol of cellobiose
GH	Glycosisde Hydrolase
EG	Endoglucanase
ESI-MS	Electrospray Ionization Mass Spectrometry
HPLC	High Performance Liquid Chromatography
HPAEC-PAD	High Performance Anion Exchange Chromatohraphy with Pulsed Amperometric Detection
HRP	Horseradish Peroxidase
LPMO	Lytic Polysaccharide Monooxygenase
MTP	Microtiter Plate
NMR	Nuclear Magnetic Resonance
PACE	Polysaccharide analysis using carbohydrate gel electrophoresis
PASC	Phosphoric Acid Swollen Cellulose
PCS	Pretreated Corn Stover
PGC	Porous Graphitized Carbon
PDH	Pyranose Dehydrogenase
PQQ	Pyrroloquinoline Quinone
PHP	Phenolphtalein
PV	Pyrocathecol Violet
RBBR	Remazol Brilliant Blue
rPHP	reduced Phenolphtalein
RP-UHPLC	Reverse Phase-Ultra High Performance Liquid Chromatography
XPS	X-ray Photoelectron Spectroscopy
